# Variation of dengue NS1 antigen measured by commercial ELISA kit in various forms of dengue infections and assessment of the association between NS1 antigen level and disease severity

**Published:** 2011-01-10

**Authors:** Veasna Duong, Sowath Ly, Sivuth Ong, Norith Chroeung, Patrich Lorn Try, Vincent Deubel, Sirenda Vong, Philippe Buchy

**Affiliations:** 1 Institut Pasteur in Cambodia, Phnom Penh, Cambodia; 2 Kampong Cham Provincial Hospital, Paediatric department, Cambodia

## Background

Dengue is a systemic infection with a wide clinical spectrum including asymptomatic, mild undifferentiated dengue fever (DF), dengue hemorrhagic fever (DHF) and dengue with shock syndrome (DSS). Detection of dengue NS1 antigen (Ag) in acute infection is a valuable tool for early diagnosis. The purpose of this study was to evaluate the sensitivity of the Platelia NS1 Ag kit (BioRad®) for the various forms of DENV infection and assess the potential role of NS1 Ag as a marker of severity.

## Methods

We conducted the study at the Kampong Cham hospital, Cambodia in 2006 and 2007. After obtaining informed consent, we randomly collected serum samples and clinical data from dengue clinically suspected patients and also samples (at day 1 and 7 or at the appearance of fever) among some patients’ family members to identify asymptomatic dengue cases.

## Results

Among 260 confirmed dengue patients, the overall sensitivity and specificity of NS1 kit were of 57.5% and 100%, respectively. NS1 Ag test combined (no significant difference in relation with other parameters) with an in-house MAC-ELISA test significantly increased the sensitivity to over 85% (p<0.001). NS1 Ag positivity rate was significantly higher when comparing (1) DF versus DHF/DSS (p<0.001); (2) primary versus secondary infection (p=0.001); (3) patient with a viremia > 5 log versus those with lower viremia (p<0.001); (4) patients infected with DENV-1 versus the other 3 serotypes (p<0.05). In asymptomatic dengue cases, the NS1 Ag positive rate was lower than in dengue patients with samples collected (within 3 days of fever onset, p=0.002 and all days merged, p=0.053). In multivariate analysis, DHF/DSS was significantly more frequent in secondary infection (adjusted OR=6.6, p<0.01) when controlled with age, sex, day of fever onset, DENV serotype and viremia. Interestingly, severity was associated with high NS1 antigen level or DENV-1 infection (adjusted ORs: 0.21 (p=0.002) and 0.083 (p=0.006) respectively).

## Conclusion

The overall sensitivity of NS1 Ag detection kit appeared relatively low; however, sensitivity varied widely depending on virus serotypes, presence/absence of anti-dengue IgG, viremia level, disease severity or asymptomatic/symptomatic infections. Nevertheless, laboratory diagnosis improved when combining NS1 Ag and MAC-ELISA testing.

